# Outer membrane vesicles of *Pasteurella multocida* contain virulence factors

**DOI:** 10.1002/mbo3.201

**Published:** 2014-07-25

**Authors:** Miguel A Fernández-Rojas, Sergio Vaca, Magda Reyes-López, Mireya de la Garza, Francisco Aguilar-Romero, Edgar Zenteno, Edgardo Soriano-Vargas, Erasmo Negrete-Abascal

**Affiliations:** 1 Carrera de Biología, Facultad de Estudios Superiores Iztacala, UNAMAv. de los Barrios # 1, Los Reyes Iztacala, Tlalnepantla, Estado de México, 54090, México[Corresp cor1]; 2 Departamento de Biología Celular, CINVESTAV-IPNAp 14-740, México D.F., 07000, México; 3 CENID-MicrobiologíaCarr. Mexico-Toluca Km 15.5, Cuajimalpa, Mexico D.F., 05110, México; 4 Departamento de Bioquímica, Facultad de Medicina, UNAMMéxico, 04510, México; 5 Centro de Investigación y Estudios Avanzados en Salud Animal, FMVZ, UAEMToluca, 50200, Mexico

**Keywords:** Outer membrane vesicles, *Pasteurella multocida*, proteases, virulence factors, *β*-lactamase

## Abstract

*Pasteurella multocida* (Pm) is a gram-negative bacterium able to infect different animal species, including human beings. This bacterium causes economic losses to the livestock industry because of its high morbidity and mortality in animals. In this work, we report the characterization of outer membrane vesicles (OMVs) released into the culture medium by different Pm serogroups. Purified OMVs in the range of 50–300 nm were observed by electron microscopy. Serum obtained from chickens infected with Pm recognized several proteins from Pm OMVs. Additionally, rabbit antiserum directed against a secreted protease from *Actinobacillus pleuropneumoniae* recognized a similar protein in the Pm OVMs, suggesting that OMVs from these bacterial species contain common immunogenic proteins. OmpA, a multifunctional protein, was identified in OMVs from different Pm serogroups, and its concentration was twofold higher in OMVs from Pm serogroups B and D than in OMVs from other serogroups. Three outer membrane proteins were also identified: OmpH, OmpW, and transferrin-binding protein. Three bands of 65, 110, and 250 kDa with proteolytic activity were detected in Pm OMVs of serogroups A and E. Additionally, *β*-lactamase activity was detected only in OMVs from Pm 12945 Amp^r^ (serogroup A). Pm OMVs may be involved in different aspects of disease pathogenesis.

## Introduction

*Pasteurella multocida* is a gram-negative bacterium that infects different animal species and human beings. It causes many widespread infections, such as snuffles in rabbits; pneumonia in cattle, sheep, and goats; fowl cholera in chickens; and atrophic rhinitis in pigs (Carter 1967). In humans, it has been associated with diseases of the lower and the upper respiratory tracts, arthritis, osteomyelitis, meningitis, and infections due to scratches by dogs and cats, among others (Felix et al. 2003).

Based on soluble capsular polysaccharides, 5 serogroups of Pm (A, B, D, E, and F) and 16 somatic serotypes are currently differentiated, but none of them is completely restricted to a specific host (Mutters et al. 1989). The key virulence factors of Pm, which are required for a successful infection, are different types of fimbrial adhesins, such as type IV pili, filamentous hemagglutinin, and short pili, and nonfimbrial adhesins, such as OmpA, capsule, and LPS. Pm also carries other virulence determinants, such as iron acquisition proteins, siderophores, and a dermonecrotic toxin only expressed by serogroup D strains (Harper et al. 2006; Hatfaludi et al. 2010).

Gram-negative bacteria possess several secretion mechanisms including outer membrane vesicles (OMVs). OMVs can be released into the surrounding medium by commensal as well as pathogenic microorganisms (Beveridge 1999). The composition of the OMVs varies depending on the species and strain of the microorganism. The composition of the OMVs also varies when the microorganism is cultured under normal or stress conditions (Mashburn-Warren and Whiteley 2006; Schooling and Beveridge 2006; Amano et al. 2010). OMVs contain toxins, enzymes, adhesins, DNA, and other outer membrane (OM) and periplasmic virulence components (Amano et al. 2010). Based on their composition following distinct functions for OMVs have been suggested: (1) predatory functions because of their ability to merge with membranes from similar or different bacteria (Mashburn-Warren and Whiteley 2006; Mashburn-Warren et al. 2008); (2) generation of nutrients or free space at the colonization site; (3) as transport vehicles because they can contain virulence factors, DNA, antibiotics, and *β*-lactamase (Ciofu et al. 2000; Mashburn-Warren et al. 2008; Schaar et al. 2011); (4) as elements that mediate adherence by promoting the agglutination of the same bacterial species or related microorganisms; and (5) as main components of biofilms (Schooling and Beveridge 2006).

The aim of this work was to isolate OMVs from cultures of Pm to identify proteins that have been described as possible virulence factors and participate in the Pm pathogenesis.

## Materials and Methods

### Bacterial strains

The following Pm strains (ATCC) were used: 12945 (serogroup A, Amp^r^), 43017 (serogroup B); 12948 (serogroup D); 43020 (serogroup E); and C44 (a field isolate of serogroup A from rabbit) (Soriano-Vargas et al. 2012).

### Isolation of outer membrane vesicles

All bacterial strains were grown in brain heart infusion broth (BHI; BD Bioxon, Cuautitlán Izcalli, México, México) at 37°C with agitation for 24 h. Bacterial cells were harvested by centrifugation (10,000*g* for 20 min at 4°C). Culture supernatants were filtered through a 0.22 *μ*m pore size membrane (Millipore Billerica, MA, USA) to remove residual cells. OMVs were recovered by ultracentrifugation (150,000*g* for 3 h at 4°C) as described previously (Negrete-Abascal et al. 2000).

### Electron microscopy

Whole bacterial cells or OMVs were placed on carbon-and Formvar-coated copper grids, negatively stained with 1% (w/v) phosphotungstic acid, and observed with a JEM 2000 EX transmission electron microscope (Peabody, MA USA) at 80 kV (Negrete-Abascal et al. 2000).

### Electrophoresis and zymograms

Total protein (10–15 *μ*g) that was obtained from Pm vesicles was loaded by well (Bradford, 1976) and separated by electrophoresis in the presence of 5% (v/v) *β*-mercaptoethanol on a 10% (w/v) SDS polyacrylamide gel, except when the analysis was carried out using gels copolymerized with 0.1% (w/v) gelatin or with 1% (w/v) casein, as described previously (Negrete-Abascal et al. 1998). Both types of gels were stained with Coomassie brilliant blue R-250 to visualize the protein patterns or proteolytic bands.

### Immunoblotting

To identify immunogenic proteins present in Pm OMVs, samples were separated by electrophoresis and proteins were transferred to nitrocellulose membranes (Sigma, St Louis, MO) for 1 h at 400 mA according to the protocol of Towbin et al. (1979). Membranes were blocked with skim milk for 1 h at room temperature, incubated with either rabbit hyperimmune serum against an *Actinobacillus pleuropneumoniae*-purified protease (Negrete-Abascal et al. 1998) or a pool of serum samples from chickens infected with Pm. Membranes were processed as described previously by Ramón Rocha et al. (2006).

### Presence of active *β*-lactamase in OMVs from Pm

Nitrocefin disks (Becton Dickinson of México, Cuautitlán Izcalli, México) were used to detect *β*-lactamase in Pm OMVs. As a positive control, a clinical isolate of *β*-lactam-resistant strain of *Staphylococcus aureus* was used, and for the negative control, Pm strains or OMVs of *β*-lactam-sensitive bacteria were used. According to the manufacturer's instructions, red color indicated resistance to the action of penicillin or cephalosporin.

## Results and Discussion

Gram-negative bacteria secrete different components into their surrounding medium, including various virulence factors, such as toxins that damage host cells, proteases that degrade molecules involved in host defense, such as immunoglobulins and structural components of the host, or elemental molecules that capture iron, such as siderophores (Confer 2009). Bacteria are also able to release OMVs, which can carry different cargo molecules, such as proteases, toxins, hemagglutinins, and fimbriae proteins; these molecules can either be contained in the OMVs or displayed on the surface. In addition, OMVs are enriched in outer membrane proteins (Kuehn and Kesty 2005; Amano et al. 2010). In addition to being important components of biofilms, OMVs allow the microorganisms to interact with host tissues or other microorganisms in the same niche (Schooling and Beveridge 2006; Mashburn-Warren et al. 2008; Ellis and Kuehn 2010).

**Figure 1:**
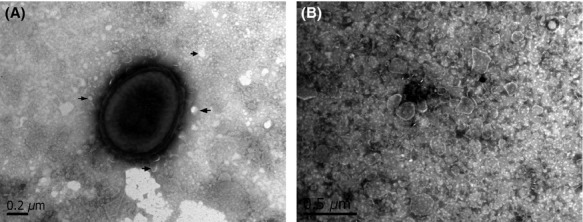
Transmission electron micrograph of negatively stained Pm 43020 strain. (A) The release of outer membrane vesicles (OMVs) from Pm can be seen in the micrograph. (B) Pm OMVs negatively stained. Arrows indicate some of the OMVs released

**Figure 2:**
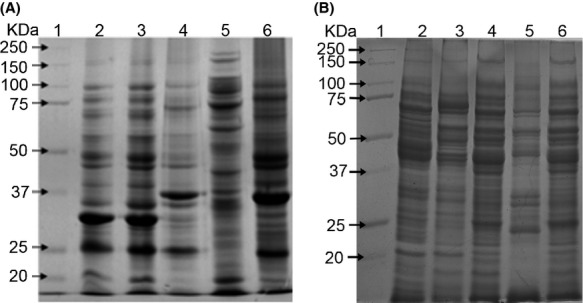
Protein pattern of outer membrane vesicles (OMVs) (A) and total cell extracts (B) from different strains of Pm as observed by electrophoresis in a 10% polyacrylamide gel. Lane 1: molecular weight markers (MWM); Lane 2: Pm 43017; Lane 3: Pm 43020; Lane 4: Pm C-44; Lane 5: Pm 12945; and Lane 6: Pm 12948. All samples were boiled in the presence of 5% *β*-mercaptoethanol.

Negative staining of released OMVs or OMVs associated with Pm bacterial surface was performed to facilitate visualization by transmission electron microscopy. OMVs varied in size from 50 to 300 nm (Fig.1A and B), with an average size of 100 nm, which is similar to the size of OMVs that have been described for *A. pleuropneumoniae*, *Avibacterium paragallinarum*, and other Gram-negative bacteria (Amano et al. 2010). No bacterial growth in OMVs samples inoculated in liquid BHI confirmed the absence of viable cells in OMVs preparations. Upon electrophoresis, the molecular weight of the proteins from OMVs was found to range from 20 to 100 kDa (Fig.2). The molecular weight range of these proteins is similar to that described for OMV proteins from *A. pleuropneumoniae* and *Av. paragallinarum*, both of which are members of the *Pasteurellaceae* family (Negrete-Abascal et al. 2000; Ramón Rocha et al. 2006).

*Pasteurella multocida* has the ability to infect a wide range of hosts. Its pathogenicity is complex, and different virulence factors, such as outer membrane proteins and porin proteins (Oma87, Psl, OmpH), type 4 fimbriae (PtfA), a filamentous hemagglutinin (PfhA), neuraminidases (NanB, NanH), iron acquisition-related factors (ExbBD-TonB, TbpA, HgbA, HgbB), a dermonecrotoxin (ToxA), and two superoxide dismutases (SodA, SodC) among others (Confer 2009; Hatfaludi et al. 2010), are involved in pathogenesis. Several of these virulence factors are located in the outer membrane and could be released in the OMVs.

**Figure 3:**
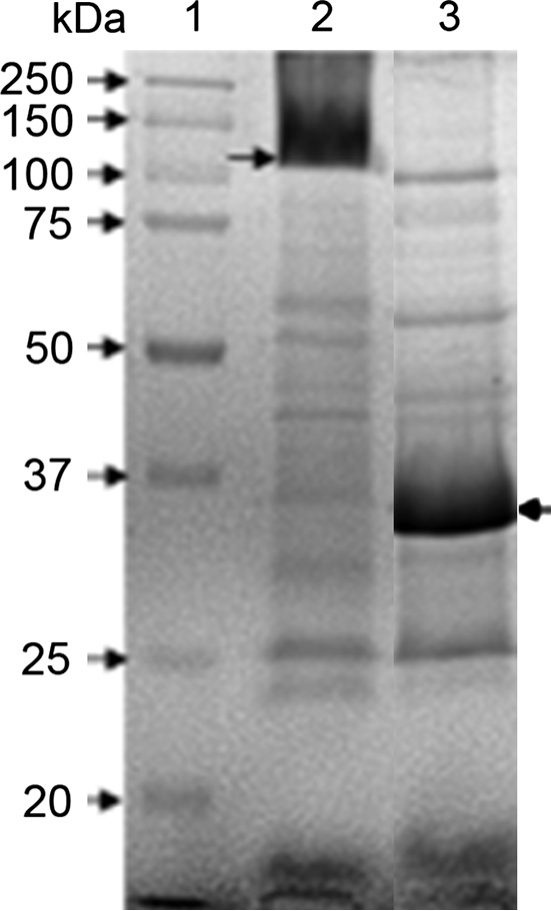
Protein patterns of outer membrane vesicles (OMVs) from Pm 12948 as observed by electrophoresis in a 10% polyacrylamide gel. Lane 1: molecular weight markers (MWM); Lane 2: sample without treatment; and Lane 3: sample was boiled in the presence of 5% *β*-mercaptoethanol. Arrows indicate the OmpA

An approximately 35–37 kDa protein was observed in OMVs from all the Pm serogroups that were studied. This protein was enriched in samples from Pm 12948 and Pm C44 strains (Fig.2). However, it migrated as a higher molecular weight protein if samples were not boiled or treated with 5% *β*-mercaptoethanol (Fig.3). Based on these properties, this protein was considered the OmpA of Pm (Hatfaludi et al. 2010). In some cases, specific proteins are enriched in OMVs while others are excluded, suggesting a specific sorting mechanism for these proteins (Kato et al. 2002).

**Figure 4:**
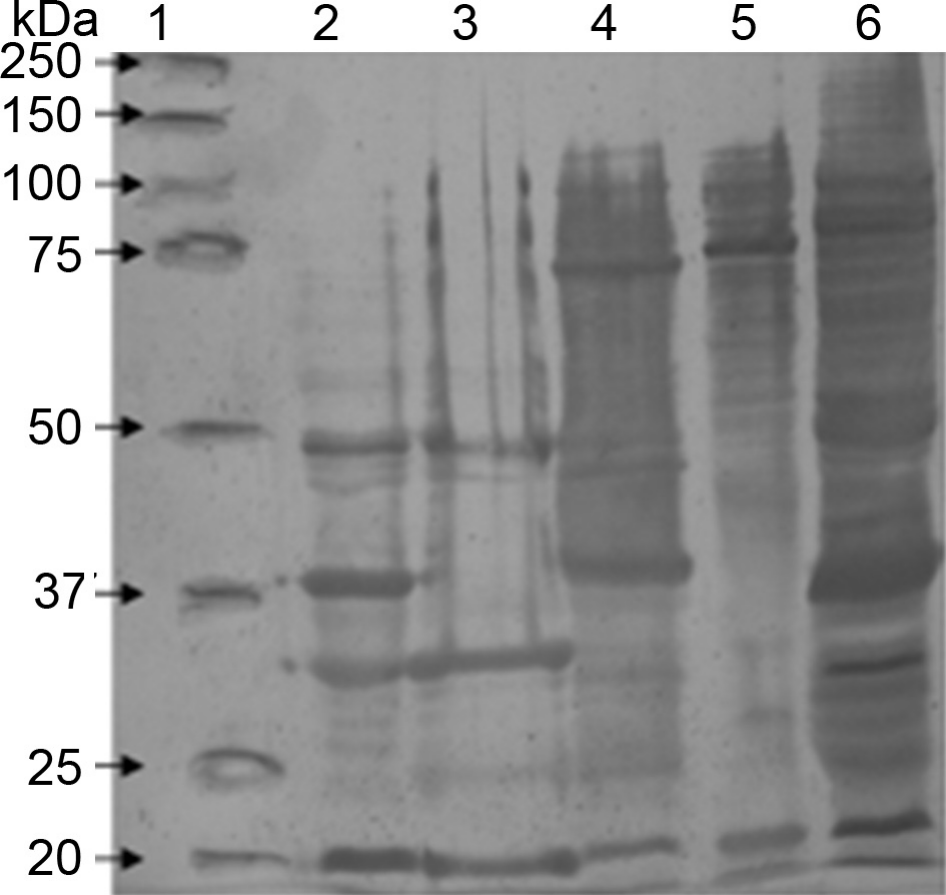
Immunorecognition of proteins from Pm OMVs using a pool of serum samples from chickens infected with Pm. The loading order and the amount of protein loaded are the same as in Figure2.

Several OMV proteins obtained from Pm were immunorecognized by serum obtained from chickens infected with Pm (Fig.4). The 35–37 kDa heat-modified protein was also recognized by serum from animals infected with *A. paragallinarum*, Pm, or *G. anatis*, suggesting the presence of a similar protein and its in vivo expression in these microorganisms (data not shown).

Due to its enrichment in OMVs, immunogenicity, and presence in different microorganisms, the 35–37 kDa protein from Pm 12948 was cut from the gel and submitted for mass spectrophotometric analysis. Six peptides were identified by in silico analysis as part of the outer membrane protein A (OmpA) from Pm and other microorganisms (Table1). This protein was characterized previously in Pm, and in addition to other functions, it is also involved in serum resistance and adhesion to host cells because of its interaction with heparin and fibronectin (Smith et al. 2007; Dabo et al. 2008; Hatfaludi et al. 2010). In *Escherichia coli*, OmpA is considered a multifunctional protein that exhibits both phage receptor activity and some porin activity; it maintains the integrity of the OM and is one of the major factors responsible for *E. coli* invasion of human brain microvascular endothelial cells (Smith et al. 2007). OmpA participates in biofilm formation and in the evasion of the immune system, and it is considered a target for development of vaccines. In addition, OmpA in *Acinetobacter baumannii*, an important nosocomial pathogen that causes a variety of human infections in critically ill patients, modulates the biogenesis and most likely the content of OMVs (Moon et al. 2012). OmpH, OmpW, and transferrin-binding protein were also identified into those outer membrane proteins immunorecognized by the sera used (Table1). OmpH is an outer membrane porin protein abundantly expressed in the *P. multocida* bacterial surface (Tan et al. 2010). OmpW is a scarcely studied protein in Pm; however, in *E. coli* this protein is important for avoiding the phagocytosis and its up-expression correlates with an increased bacterial survival during phagocytosis (Wu et al. 2013). This protein could be important for Pm virulence. Transferrin-binding proteins are considered virulence factors in several microorganisms, since they participate in iron uptake.

**Table 1:** Peptide mass fingerprint of different immunogenic proteins from Pm OMVs.

	*m*/*z*	Residue	Sequence
OmpA	1871.7	42–59	ASHDLGEGLSALAYAELR
	2142.7	143–163	SAEFNGFTFGGATVFSAGADK
	1094.5	171–180	GFVVAGLYNR
	2381.9	182–203	NGDVGFALEAGYSQEYVTETAK
	1108.5	254–262	VYTDLIWAK
	1284.5	286–296	QVETFVEGGYR
OmpH	933.4	29–37	GDLVDNGSR
	1873.9	42–59	ASHDLGEGLSALAYAELR
	2201.4	138–158	SAEFNGFTFGGAYVFSADADK
	1095.6	166–175	GFVVAGLYNR
	2250.0	177–197	MGDVGFALEAGYSQKYVTVAK
	1464.4	233–245	ALEVGLNYDINDK
OmpW	1866.1	31–47	GGPILVVPNASTNHDVFK
	2082.9	93–109	TKHLPPSLYAQYYFLDK
	2169.4	95–112	HLPPSLYAQYYFLDKDAK
	2007.2	113–130	ARPYVGAGVNYTTFFSEK
	1028.9	130–140	AVLNGVTDLK
	1483.3	191–203	LDPTVFFVGLGYR
Tbp	2032.3	116–136	VAVIVDGIPQAESTMSTSAR
	1945.5	142–158	HNGNINNIEYENVSSLK
	1884.2	251–266	GKPNPLNYYTTSWLTK
	1701.4	351–365	NKLDSTMSFVYLQR
	2758.6	526–549	TSSQFLPNPDLQPETALNHEISYR
	1650.7	718–731	NVILNMGVFNLFNR

Data about virulence genes have been reported mainly for two of the five strains studied here: Pm 12948 and Pm 12945 (Verma et al., 2013; Ewers et al. 2006).

**Figure 5:**
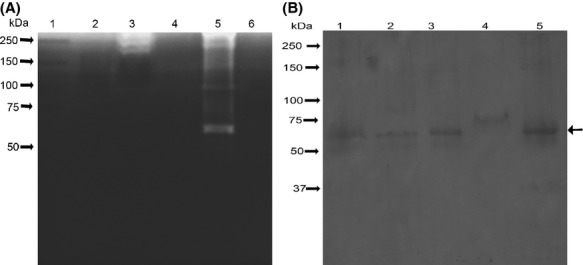
(A) Proteolytic activity of Pm outer membrane vesicles (OMVs) in 10% polyacrylamide gel copolymerized with 0.1% porcine gelatin. The loading order and the amount of sample loaded are the same as in Figure2. (B) Immunorecognition of putative proteases in Pm OMV samples using *Actinobacillus pleuropneumoniae* antiprotease serum. Lane 1: secreted proteins of *A. pleuropneumoniae*; Lane 2: Pm 43017; Lane 3: Pm 43020; Lane 4: Pm 12945; and Lane 5: Pm C-44. Arrow indicates the main band of recognition.

Zymograms using porcine gelatin or casein as substrates revealed 65, 110, and 250 kDa bands of proteolytic activity in OMV samples prepared from Pm 43020 and Pm C44 (Fig.5A). The proteolytic activity was inactivated in the presence of 20 mmol/L EDTA, which is a characteristic of metalloproteases that have been previously described in field isolates of Pm from several animal species. These metalloproteases have been shown to degrade IgG molecules (Negrete-Abascal et al. 1999). Although only OMV samples from Pm 43020 and Pm C44 exhibited proteolytic activity, bands of approximately 60 kDa were immunorecognized by a rabbit polyclonal serum (raised against the purified protease from *A. pleuropneumoniae*) in OMV samples from all the Pm strains that were examined (Fig.5B), a similar recognition band was observed in a sample of *A. pleuropneumoniae* secreted proteins used as positive control of recognition. This immunorecognition corresponds to a band of proteolytic activity observed in the zymogram (Fig.5A). This result suggests that proteases are associated with OMVs in both active and inactive forms. The association of inactive proteases with OMVs has been described for *A. pleuropneumoniae* and *A. paragallinarum* (Negrete-Abascal et al. 2000; Ramón Rocha et al. 2006).

**Figure 6:**
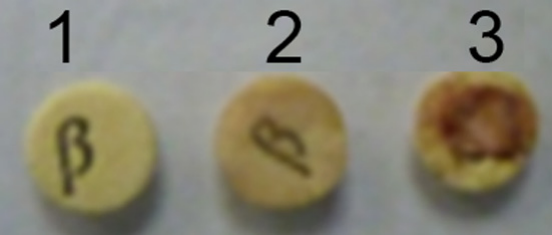
Detection of *β*-lactamase associated with outer membrane vesicles (OMVs) using nitrocefin disk. (1) Penicillin-sensitive Pm 12948 strain; (2) OMVs; and (3) penicillin-resistant Pm 12945 cells.

Of all the Pm strains tested for *β*-lactamase, only Pm 12945 was positive. This result is in good agreement with a previous study performed by Sellyei et al. (2009) to test the susceptibility of *P. multocida* isolated from chickens and pigs to antimicrobial agents. In that study, the authors found that the majority of *P. multocida* strains tested were susceptible to penicillin. OMVs derived from Pm 12945 strain tested positive on nitrocefin disks but a strong response was observed when the bacterial cells were included. This confirmed the presence of active *β*-lactamase enzymes in OMVs. A negative reaction was observed when culture medium or the OMVs from *β*-lactam antibiotic-sensitive Pm strains were used instead of Pm 12945 strain (Fig.6). Releasing *β*-lactamase activity in Pm OMVs may contribute to permanence into the host although not all the bacteria in situ contain a *β*-lactamase resistance gene in their genome. It has been shown that *Moraxella catarrhalis*, a human respiratory pathogen, releases OMVs containing active *β*-lactamase, which can rescue other amoxicillin-sensitive respiratory pathogens, including *M. catarrhalis*, *Streptococcus pneumoniae*, or *Haemophilus influenzae* (Schaar et al. 2011) from *β*-lactam killing. Presence of *β*-lactamase has also been described in *Pseudomonas aeruginosa* OMVs (Ciofu et al. 2000). In order to know if *β*-lactamase was a contamination from cell debris, OMVs were treated with proteinase K, to digest *β*-lactamase associated with the outer membrane of the OMVs, and permeabilized with 0.02% Triton X-100 and *β*-lactamase activity was measured on nitrocefin disks as before. Although activity diminished highly, it does not disappear, suggesting that *β*-lactamase activity was present externally and inside of OMVs (data not shown).

These results demonstrate that the composition of OMVs is complex and distinct from the OM, and accentuates the potential for a diversity of biological functions for these vesicles. The different virulence factors in OMVs released by a pathogen could be responsible for damage caused during host infection. As with other microorganisms, they could also be good immunogens as components of vaccines.
